# Increased Cycling Cell Numbers and Stem Cell Associated Proteins as Potential Biomarkers for High Grade Human Papillomavirus+ve Pre-Neoplastic Cervical Disease

**DOI:** 10.1371/journal.pone.0115379

**Published:** 2014-12-22

**Authors:** Maurice Canham, Chara Charsou, June Stewart, Sharon Moncur, Laura Hoodless, Ramya Bhatia, Duanduan Cong, Heather Cubie, Camille Busby-Earle, Alistair Williams, Victoria McLoughlin, John D. M. Campbell, Kate Cuschieri, Sarah Howie

**Affiliations:** 1 Human Papillomavirus Group, University of Edinburgh, Edinburgh, United Kingdom; 2 Medical Research Council Centre for Regenerative Medicine, University of Edinburgh, Edinburgh, United Kingdom; 3 Medical Research Council Centre for Inflammation Research, University of Edinburgh, Edinburgh, United Kingdom; 4 Simpson Centre for Reproductive Health, Royal Infirmary of Edinburgh, Edinburgh, United Kingdom; 5 Simpson Centre for Reproductive Health, University of Edinburgh, Edinburgh, United Kingdom; 6 Scottish National Blood Transfusion Service National Science Laboratory, Edinburgh, United Kingdom; 7 Scottish Human Papillomavirus Reference Laboratory, Royal Infirmary of Edinburgh, Edinburgh, United Kingdom; Georgetown University, United States of America

## Abstract

High risk (oncogenic) human papillomavirus (HPV) infection causes cervical cancer. Infections are common but most clear naturally. Persistent infection can progress to cancer. Pre-neoplastic disease (cervical intraepithelial neoplasia/CIN) is classified by histology (CIN1-3) according to severity. Cervical abnormalities are screened for by cytology and/or detection of high risk HPV but both methods are imperfect for prediction of which women need treatment. There is a need to understand the host virus interactions that lead to different disease outcomes and to develop biomarker tests for accurate triage of infected women. As cancer is increasingly presumed to develop from proliferative, tumour initiating, cancer stem cells (CSCs), and as other oncogenic viruses induce stem cell associated gene expression, we evaluated whether presence of mRNA (detected by qRT-PCR) or proteins (detected by flow cytometry and antibody based proteomic microarray) from stem cell associated genes and/or increased cell proliferation (detected by flow cytometry) could be detected in well-characterised, routinely collected cervical samples from high risk HPV+ve women. Both cytology and histology results were available for most samples with moderate to high grade abnormality. We found that stem cell associated proteins including human chorionic gonadotropin, the oncogene TP63 and the transcription factor SOX2 were upregulated in samples from women with CIN3 and that the stem cell related, cell surface, protein podocalyxin was detectable on cells in samples from a subset of women with CIN3. SOX2, TP63 and human gonadotrophin mRNAs were upregulated in high grade disease. Immunohistochemistry showed that SOX2 and TP63 proteins clearly delineated tumour cells in invasive squamous cervical cancer. Samples from women with CIN3 showed increased proliferating cells. We believe that these markers may be of use to develop triage tests for women with high grade cervical abnormality to distinguish those who may progress to cancer from those who may be treated more conservatively.

## Introduction

The cancer stem cell (CSC) hypothesis [1] suggests that the bulk of transformed cells within cancers have limited proliferative capacity and arise from a relatively small fraction of CSCs which are capable of unlimited self-renewal. The CSCs are thought to initiate the original tumour and any metastatic tumours. CSCs were first identified in acute myeloid leukaemia [2] and have since been shown in many solid tumours including melanoma [3], [4] and breast [5], pancreatic [6], lung [7], head and neck [8], brain [9], colon [10], [11], prostate [12], and liver [13], [14] cancers. CSCs often have complex phenotypes but have been characterised by their expression of “stemness genes” including NANOG (OMIM: 607937), OCT4 (OMIM: 164177), SOX2 (OMIM: 184429) and podocalyxin (OMIM: 602632) [15]–[18]. In virally induced cancers the action of viral proteins on host tissue cells may alter expression of “stemness” genes and promote CSC development. Hepatitis C virus infection has been reported to induce CSCs in human liver [19] and the major EBV oncogene LMP1 was reported to induce a cancer stem cell phenotype in nasopharyngeal epithelial cells [20]. Up-regulation of “stemness” gene protein expression in cancer can thus serve as an indicator of neoplastic change.

Worldwide, nearly all cervical carcinomas are caused by known high risk (oncogenic) types of Human Papilloma Virus (HR-HPVs) with the majority attributed to persistent infection by HPV-16 or HPV-18 [21]. Cervical cancer and its precursor stages of cervical intraepithelial neoplasia/CIN (graded 1–3 according to severity) typically occur in cells of the transformation zone where columnar epithelium undergoes metaplastic change into squamous epithelium. There is a need to better understand the host - virus interactions that lead to different disease outcomes and to develop adjunct biomarker tests for more accurate triage of infected women. CIN2 or worse is currently regarded as the standard threshold for treatment of pre-neoplastic lesions. However, as treatment itself carries a level of morbidity, it is of interest to determine more accurately which women are at high risk of cancer development and which women might be managed more conservatively.

Where implemented, cervical cancer screening programmes have reduced the incidence of invasive disease by recognising and treating pre-invasive lesions. Nevertheless there is still room for improvement, particularly in the selection of cases which require treatment. Although most screening programmes involve cytological examination of exfoliated cervical epithelial cells, molecular HPV testing is being widely introduced to enhance sensitivity. A paradigm shift is imminent whereby the primary screening modality for the future is likely to be HPV testing. However, one significant issue even with clinically validated HPV tests is their inability to determine which infections will result in significant disease. Further, while the clinical sensitivity of HPV testing consistently exceeds that of cytology, the specificity is less optimal. Thus there is a need to develop adjunct triage tests for biomarkers that are more specific for detection of HPV related high grade disease. To this end, we investigated whether there was an association of CSC markers and/or cell proliferation that would differentiate HR-HPV+ve high grade pre-neoplastic cervical disease from insignificant lesions in cervical samples routinely taken for liquid based cytology (LBC) and for which biopsy data was available. We further investigated expression of stem cell related proteins in biopsies of HPV+ve squamous carcinoma of the cervix.

## Materials and Methods

### Ethics statement

Ethical approval was obtained from Scotland A Research Ethics Committee (REF 12/SS/0034). All cervical samples were collected into ThinPrep-preservcyt liquid based cytology transport medium (Hologic, Crawley, UK). For this study, 95 anonymised, curated, cervical smear samples were obtained from the Scottish National HPV archive, which holds Generic Scotland A Research Ethics Committee approval for Research Tissue banks (REC Ref 11/AL/0174) for provision of samples for HPV related research after approval from an Independent Scottish HPV Archive Steering Committee. For this study our HPV Archive Application was Reference 0004. The cervical samples used in this study were obtained from different collections within the Scottish HPV Archive. Cytology normal samples were from residual samples from National HPV Surveillance Programme (Opt-out consent through the screening programme approved by Tayside Research Ethics Committee Ref 11/AL/0174) and the abnormals from two previously collected research projects (EAS, Scotland A Research Ethics Committee Ref 07/S0501/92, and COHGS Scotland A Research Ethics Committee Ref 09/S0801/106). Additionally, sections from 10 anonymised, formalin fixed cervical biopsies from women with SCCC and 10 normal cervical biopsies from women who had undergone hysterectomy for benign reasons were obtained from the Royal Infirmary of Edinburgh Pathology Department Archive, via the South East Scotland (Lothian) SAHSC Bioresource. The NHS Lothian SAHSC Bioresource holds RTB approval from the East of Scotland REC, (reference number 13/ES/0126) and provides samples with approval of the Tissue Governance Committee.

### Samples

For most samples with abnormalities, information on both cytology and histology was available (see Table 1 for details). In addition, sections from 10 anonymised, formalin fixed cervical biopsies from women with SCCC and 10 normal cervical biopsies from women who had undergone hysterectomy for benign reasons were obtained from the Royal Infirmary of Edinburgh Pathology Department Archive, via the East of Scotland Bioresource.

**Table 1:** Disease status, age range and cytology results for cervical LBC samples obtained from the Scottish national HPV Archive.

HPV & Disease (cytology/biopsy) Status	No.	Age in years		Cytology result, dyskaryosis				
		range	median	None n =	mild n =	moderate n =	severe n =	unknown n =
HPV -ve cyto -ve	28	20–34	20	28	0	0	0	0
HR-HPV +ve cyto -ve	9	20–43	20	9	0	0	0	0
HR-HPV +ve CIN 1	13	22–40	23	1	6	0	0	7
HR-HPV +ve CIN 2	22	20–28	23	0	12	6	1	3
HR-HPV +ve CIN 3	23	20–25	23	0	0	3	17	2

### HPV Genotyping

All cervical samples were genotyped for HPV status. Automated extraction of DNA from LBC samples was performed used the MDX media Kit (Qiagen, Manchester, UK). DNA was extracted from 10µm sections cut from the biopsies using QIAamp DNA Mini Kit (Qiagen, UK) following the manufacturer's instructions. As we have previously published [22], genotyping was performed using the Multimetrix HPV Assay (Diamex, Heidelberg, DE). This assay is based on luminex technology and is capable of detecting 18 high-risk or putatively high-risk types and 5 low-risk types according to the current IARC classification. Among the HR-HPV+ve cervical samples or biopsies, 69% overall contained either or both types 16 and 18. All 10 biopsies from women with SCCC were HR-HPV+ve; of the 10 cervical biopsies from women who had undergone hysterectomy for benign reasons, 8 were HPV-ve and 2 were HR-HPV+ve. All LBC samples from women with CIN1, CIN2 or CIN3 were HR-HPV+ve; of the LBC samples with normal morphology, 28 were HPV-ve, and, 9 were HR-HPV+ve (Table 1).

### Proteomic array

Since the collection medium ThinPrep preservcyt contains 50% methanol we used a methanol/chloroform based protein extraction method. Briefly, 100µl ice cold chloroform was added to 900µl of sample in an Eppendorf tube on ice, vortexed, incubated on ice for 5min and spun for 5min at 10,000rpm at 4°C in a benchtop microfuge. The aqueous top layer was discarded, 300µl methanol was added and the tube vortexed, and spun for 10 min at 10,000rpm at 4°C in a benchtop microfuge. The supernatant was removed and the pellet resuspended in 100µl Tris Buffered Saline. Protein concentration was determined by Pierce BCA assay (Thermo Scientific, UK). Antibody-based, pluripotent stem cell proteomic arrays (Cat No. ARY010, R&D Systems, UK) were carried out as per manufacturer's instructions. To identify proteins of interest, for the arrays, pools of samples were compared. Each pool contained 10µg of protein from each individual sample (see Results). Developed arrays were scanned and pixel density calculated using Image-J software.

### Flow cytometry

A) Staining protocol: -1 ml aliquots from LBC samples in Preservcyt were passed through a BD Falcon filter-top 5 ml FACS tube (BD Biosciences), to produce a filtered cell suspension. This was then centrifuged at 300×g in a bench top microfuge, the supernatant removed, and the pellet re-suspended in 400µl MACSQuant running buffer (Miltenyi Biotec Ltd, UK) (“buffer”). 200µl of cell suspension was stained with either isotype control (PE—IgM), or anti-TRA-1-60-PE (both Miltenyi Biotec). Additionally, 5µl of the DNA chelating fluorescent dye, DRAQ5 (Biostatus, UK) at 1∶20 dilution was added to all samples. Samples were incubated for 15 minutes at room temperature in the dark and then directly analysed (with no further washing) using a MACSQuant Vyb or MACSQuant 10 flow cytometer (Miltenyi Biotec). The entire 200µl of sample was acquired per tube. Samples were analysed using MACSQuantify software v2.4. Area and height were collected for all parameters, antibody stained samples were acquired on a hyper log (bi-exponential) scale, and DRAQ5 was acquired on a linear scale.

**Figure 1:**
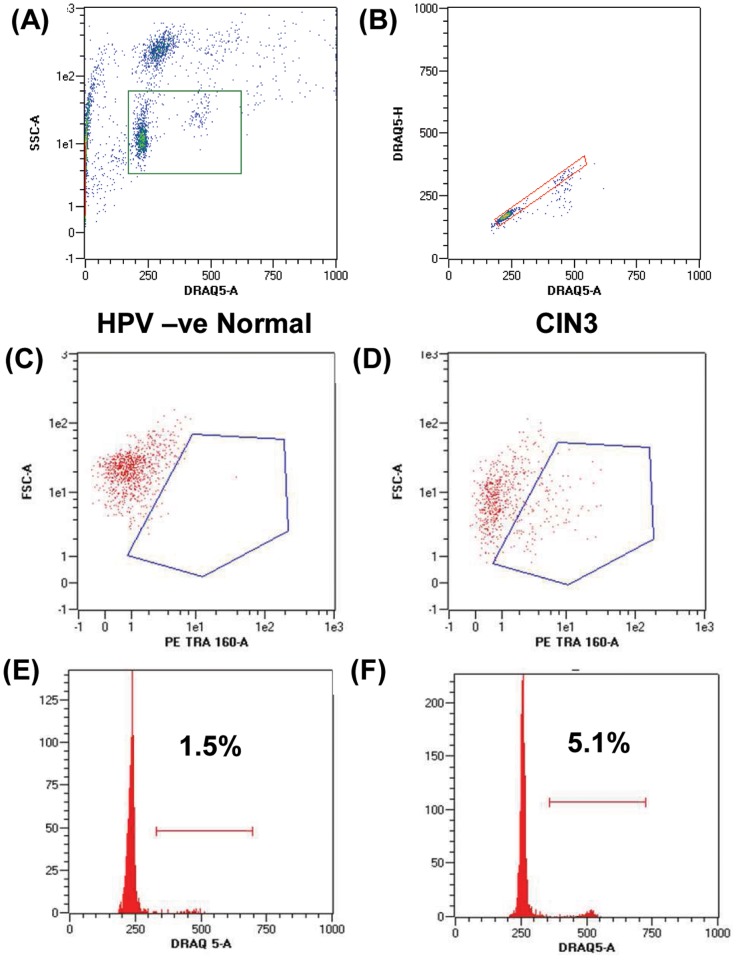
Sample Flow Cytometry analysis showing gating strategy. Debris (no DNA stain) and cell clumps (high SSC) are excluded from the analysis (A). Doublets excluded by plotting DRAQ5 area versus height (B). (C) and (D), Cells in HPV-ve normal samples do not stain with anti-TRA-1-60 but strong staining is seen in some samples from women with CIN3 (gates set against individual sample isotype controls). (E) and (F), representative cell cycle profiles from an HPV-ve normal sample (E) and a sample from a patient with CIN3 (F), showing an increased G2/M peak in (F).

B) Analysis protocol: - Fig. 1 shows representative histograms outlining the gating strategy for analysis. As samples contained variable amounts of debris, initial gating was carried out using an SSC versus DRAQ5 gate to exclude any events which did not contain nuclear material (Fig. 1A). Doublets were then excluded from the analysis by gating DRAQ5 area versus height (Fig. 1B). The singlet gate was then used to generate an SSC vs. PE plot (Fig. 1C&D) and a DNA histogram for cell cycle analysis (Fig. 1E&F). Due to variability in the material analysed, DNA histograms were analysed as a simplified percentage of events in the G0/G1peak versus the S/G2/M peak. Positive staining with TRA-1-60-PE was determined after subtracting the appropriate isotype background.

### qRT-PCR for SOX2, HCG and TP63

RNA was extracted from 1.5 ml aliquots of LBC samples using miRNeasy mini kit (Qiagen, UK) following the manufacturer's instructions. RNA was quantified on a Nanodrop spectrophotometer and stored at −80°C until use. cDNA was made from 500 ng RNA using the Quantitect Reverse Transcription Kit (Qiagen, UK) as per manufacturer's instructions and stored at −20°C until use. Duplex qPCR was carried out in Lightcycler Nano (Roche) realtime PCR system in duplicate in 8 well PCR strips using plates, primers, probes and reagents from Life Technologies Applied Biosystems, UK. Wells contained 2µl cDNA, 1µl gene of interest primer mix, 0.12µl each of 18S forward and reverse primers, 1.6µl 18S probe, and 13µl mastermix. The PCR programme was 2 min at 50°C, 10 min at 95°C, followed by 40 cycles of 15 sec at 95°C and 1 min at 60°C.

### Immunohistochemistry

Sections (4µm) from formalin fixed, paraffin embedded cervical biopsies were dewaxed in xylene and rehydrated through alcohol to water. Antigen retrieval was performed in 0.9 M citrate buffer in a pressure cooker in an 800W microwave oven for 15 minutes at full power. After cooling, slides were blocked with avidin-biotin block followed by protein block (Insight Biotechnology Ltd, UK) and stained with goat-anti-SOX2 antibody (R&D Systems, UK) or mouse monoclonal anti-TP63 antibody (AbCam, UK) overnight at 4°C. Sections were then washed, incubated with rabbit polyclonal biotinylated anti-goat or anti-mouse IgG (Vector Labs, UK), washed, incubated with streptavidin-HRP, washed and colour developed using DAB solution (Vector Labs). Sections stained with only secondary antibody (no primary control) were included in each run. Parallel sections from 11 cases were stained with SOX2 and TP63. Tumour cells were evaluated for their nuclear expression of the transcription factors. There was no significant difference between TP63 and SOX2 levels (Wilcoxon ranked pairs test). For each section stained, five fields were photographed at ×200 magnification. Using the ImageJ software “cell counter” plug-in, each picture was overlaid with a grid and nuclear +ve and –ve tumour cells counted. At least 150 cells per field (750 per section) were counted.

### Statistical analysis

Data were analysed with Graphpad Prism software. The Kruskall Wallis test with Dunn's multiple comparison post-test was used to compare normal and all disease grade groups. To compare two groups the Mann-Whitney test or Wilcoxon Ranked Pairs test was used as appropriate. Significance was assumed if p was <0.05.

## Results

### Proteins associated with cancer stem cells can be detected in LBC samples from patients with CIN3

**Figure 2:**
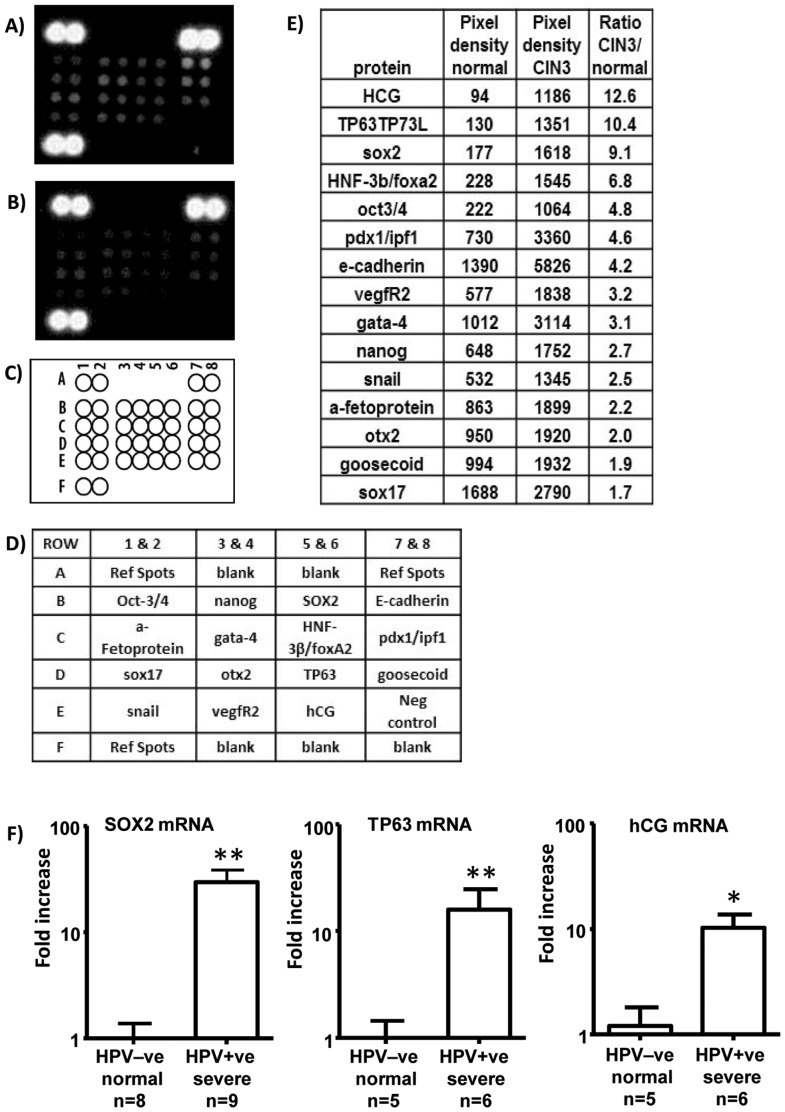
Proteomic array detection of stem cell associated proteins in pooled aliquots from 9 HR-HPV+ve cervical samples from women with CIN3 (A) and 9 HPV –ve samples with normal cytology (B); (C & D) map and key for array spots; (E) average pixel density ranked by ratio of pooled CIN3:normal samples for each protein on the array; (F) sox2, TP63 and HCG mRNAs are upregulated in HPV+ve cervical samples with severe dyskaryosis compared to HPV–ve samples with normal morphology (* = p<0.05, ** p<0.01, Mann Whitney test).

To determine whether cancer stem cell related proteins were detectable in LBC samples, we used a proteomic microarray for human stem cell proteins. The arrays were carried out on pooled HR-HPV –ve samples from 9 women with no disease and on pooled HR-HPV+ve samples from 9 women with CIN3. Arrays were developed as described in Materials and Methods and pixel density of individual spots analysed on inverted images (Fig. 2) with Image-J software. A number of proteins including HCG, TP63 and SOX-2 were upregulated in the pooled samples from patients with CIN3 compared to the pooled samples with normal morphology. To validate this finding further samples that were either cytologically normal and HR-HPV-ve, or HR-HPV+ve with severe dyskaryosis, were individually evaluated for SOX2, TP63 and HCG mRNA by qRT-PCR. All three mRNAs were elevated in samples with severe dyskaryosis (Fig. 2).

### Flow cytometry shows increased TRA-1-60+ve cells in LBC samples from patients with CIN3

**Figure 3:**
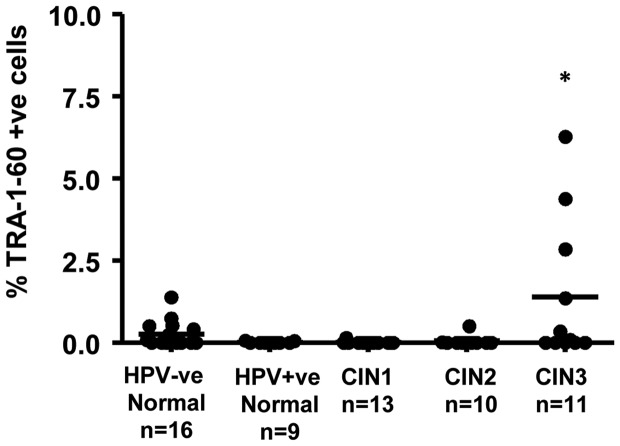
Cervical samples from women with CIN3 have increased numbers of TRA-1-60+ve cells detected by flow cytometry (see Materials and Methods for details), 1 way ANOVA (Kruskall Wallis test with Dunn's post-test versus HPV+ve normal group), * =  p<0.05.

We next investigated whether flow cytometry would be a practical methodology for the detection of markers associated with cancer stem cells in cervical samples. Most of these proteins are intracellular and preliminary attempts to stain for NANOG and OCT4 revealed that background staining is a problem with intracellular proteins (not shown) in these samples. For this reason, we concentrated on detection of the cell surface carbohydrate epitope of podocalyxin detected by the antibody TRA-1-60. Fig. 3 shows that overall, LBC samples from patients with HR-HPV+ve CIN3 s had a small but significant increase in the number TRA-1-60+ve cells compared to LBC samples with normal morphology that were either HR-HPV +ve or -ve.

### Flow cytometry indicates increased numbers of cycling cells in LBC samples with high grade disease stratified by either biopsy or cytology result

**Figure 4:**
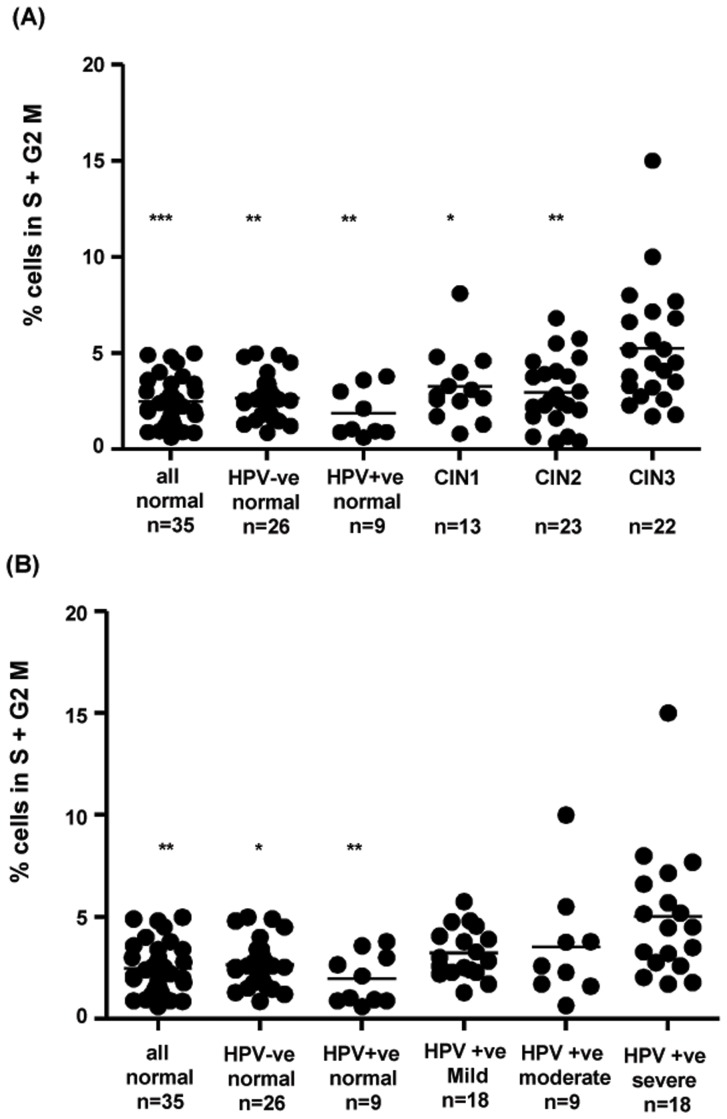
Flow cytometric detection of cycling cells in cervical samples. (A) LBC samples stratified by HPV status and histology; samples from women with CIN3 are significantly different from samples with normal cytology and from CIN1 and CIN2, 1 way ANOVA (Kruskall Wallis test with Dunn's post-test versus CIN3 group); (B) LBC samples stratified by HPV status and cytology results only; samples with severe dyskaryosis are significantly different from all normal samples 1 way ANOVA (Kruskall Wallis test with Dunn's post-test versus severe disease group). * p = <0.05, ** p = <0.01, *** p = <0.001.

Cell cycle was detected by analysing staining with the DNA chelating dye DRAQ5. Cycling cells were determined to be in the S+G2M phase of the cell cycle as described in Materials and Methods. No aneuploid cells were detected in any samples and cell cycle stages were comparable between samples. Fig. 4 shows that significant increases were seen in cervical samples from women with significant disease compared to normal samples regardless of whether normal samples were HPV-ve or HPV+ve and regardless of whether significant disease was stratified as biopsy proven CIN3 (Fig. 4A) or by cytology determined severe dyskaryosis (Fig. 4B).

### Stem cell marker expression is further increased in HR-HPV+ve LBC samples from patients with CIN3 that had the highest numbers TRA-1-60+ve cells

**Figure 5:**
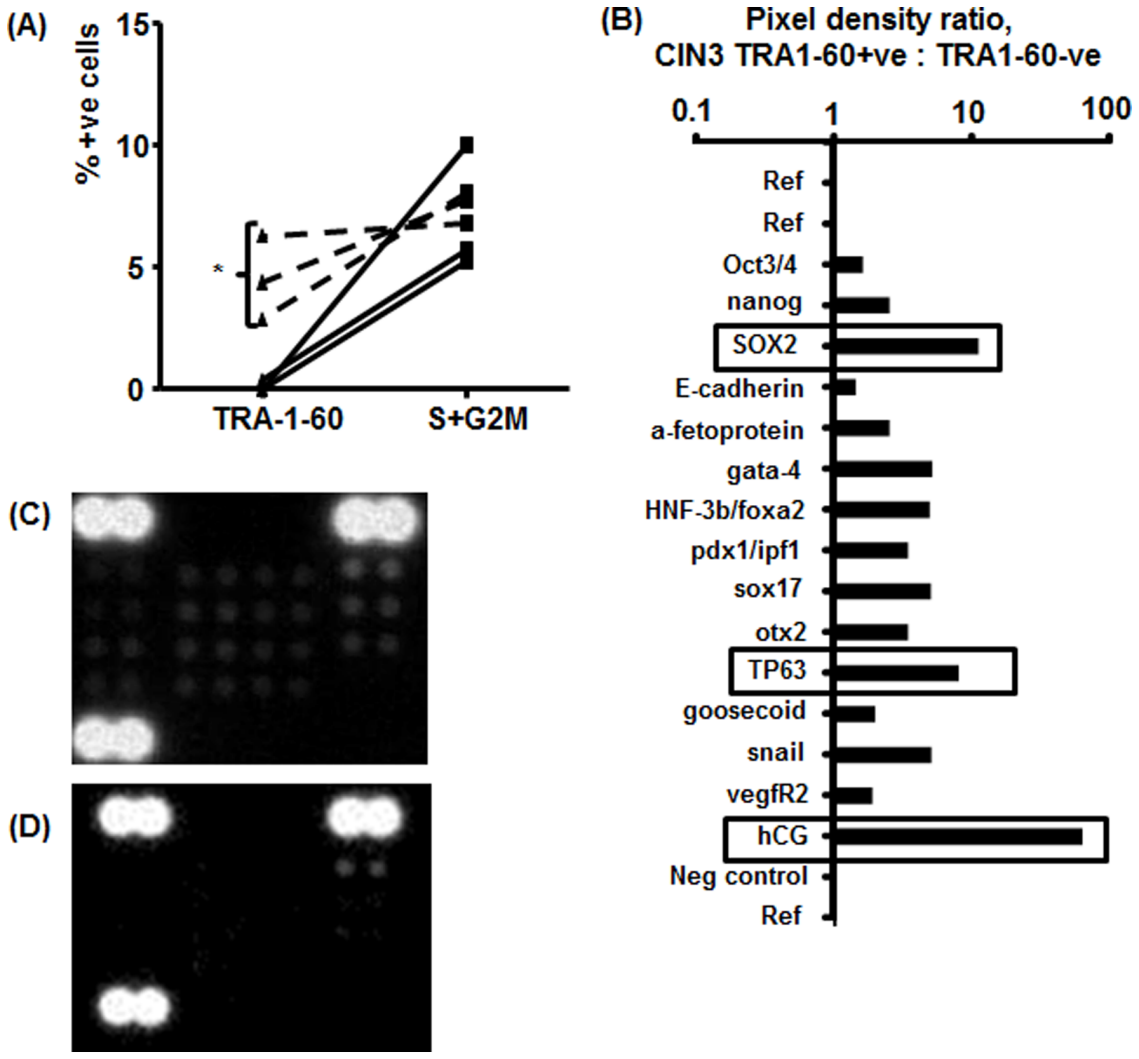
Samples with increased TRA-1-60+ve cells from patients with CIN3 have enhanced expression of stem cell proteins. A) Flow cytometric analysis of samples used for proteomic microarray. Dotted lines show that the three samples with a significant increase in TRA-1-60 +ve cells have similar levels of cycling cells to the other three CIN3 samples. *  = p<.05. B) Image-J pixel density analysis of stem cell proteomic microarray. SOX2, TP63 and HCG are further increased in samples with increased TRA-1-60+ve cells. C) Inverted image of the scan of the array from TRA1-60+ve samples; D) Inverted image of the scan of the array from TRA1-60 –ve samples.

To determine whether or not the 3 samples with the highest number of TRA-1-60+ve cells represented a subset of the CIN3 cases with potentially higher cancer stem cell presence, an equal amount of protein from each of these samples was pooled and compared to an equivalent pool of proteins from 3 samples with HR-HPV+ve CIN3 that showed no increase in TRA-1-60 using the same proteomic array. Flow cytometry results for the samples selected are shown in Fig. 5a. The three cases with increased TRA-1-60 +ve cells show similar numbers of cycling cells to the other CIN3 cases used for this analysis. Fig. 5b shows that there was an enhancement of signal for HCG, TP63 and SOX2 when the samples from the CIN3 cases with increased TRA-1-60+ve cells were compared to the other CIN3 cases.

### SOX2 and TP63 are strongly expressed in tumour cells in HR-HPV+ve cervical cancer biopsies

**Figure 6:**
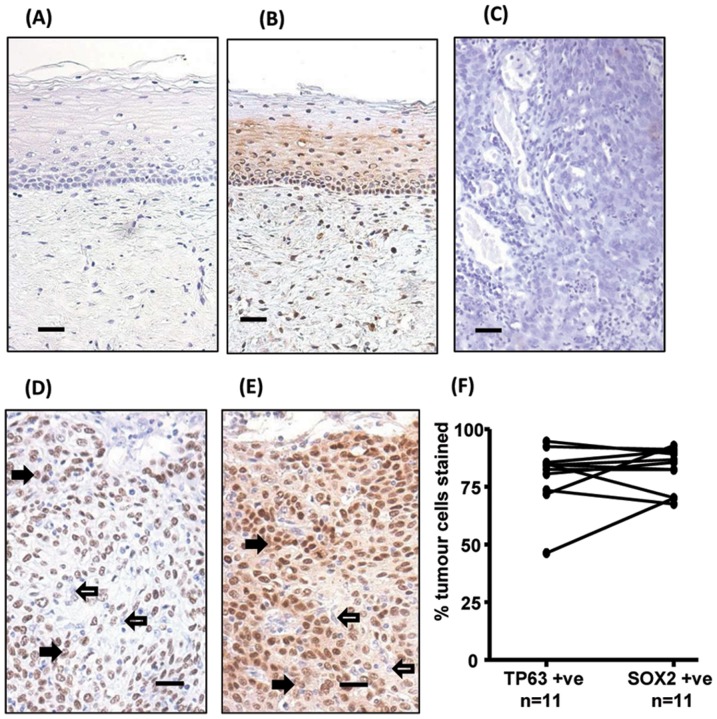
TP63 and SOX2 staining in cervical biopsies. Immunohistochemical staining of cervical biopsies. Bars  =  50µm.(A) normal cervix no primary control; (B) normal cervix stained with anti-SOX2; (C) Squamous cell cervical carcinoma no primary control; (D) representative TP63, and (E) representative SOX2, staining of tumour cells. For both TP63 and SOX2 staining was seen in the nucleus of positive cells (examples indicated by solid arrows); negative cells were a minority of tumour cells (examples indicated by unfilled arrows). (F) Image analysis results of % nuclear +ve tumour cells in biopsies. Parallel sections from 11 cases were stained with SOX2 and TP63. Tumour cells were evaluated for their nuclear expression of the transcription factors. There was no significant difference between the data for SOX2 and TP63 (Wilcoxon signed rank test).

To further validate the observation that enhanced SOX2 and/or TP63 protein in cervical samples, from women with high grade pre-neoplastic cervical disease, might relate to tumour progression, we stained a series of parallel sections from cervical biopsies from 11 women with HR-HPV+ve SCCC and 11 women with normal cervical tissue with anti-SOX2 and anti-TP63 antibodies. Fig. 6 shows representative staining. Both antibodies stained the basal layers of squamous epithelium in normal cervix and identified tumour cells in SCCC biopsies. Image analysis of the % of tumour cells staining revealed that the majority of tumour cells were positive for each marker and that there was no significant difference (p = 0.83) between the % cells stained with SOX2 (83.7±3.9) and the % cells stained with TP63 (80.2±3.9) (Fig. 6).

## Discussion

Up-regulation of hCG is reported in a number of cancers including SCCC [23] and is associated with poor prognosis [24]. TP63 is a homologue of the tumour suppressor protein TP53. In adult epithelial stem cells TP63 is a master regulator for the proliferative potential of these cells [25] and interacts with SOX2 in squamous cell carcinomas [26]. The detection of TP63 in proliferating basal squamous cells has been noted in various cancers [27] including breast [28], prostate [29], and cervical cancer [30], [31]. TP63 has also been implicated as a biomarker of cervical disease progression [32].

SOX2 is a core transcriptional regulator of pluripotency in mouse and human embryonic stem cells [33] and its transient expression together with OCT4, KLF4 and C-MYC in adult somatic cells is sufficient to reverse the developmental clock, to generate reprogrammed induced pluripotent stem cells [34]. Following embryonic development, SOX2 has also been shown to be required for the maintenance of adult stem cells residing in various tissues of the body including the gut [35], the skin [36], the eye lens and the cervix [37]. SOX2 is important in epithelial cell differentiation and is expressed in basal epithelium of the tongue [38] and in tongue squamous cell carcinoma [39]. Gene analysis has identified SOX2 and TP63 gene expression as important in defining molecular subtypes in HPV related head and neck cancer [40] and SOX2 has been associated with a number of other human cancers [41]–[43] including cervical cancer [44]. Recently, in a murine skin cancer model SOX2 was shown to be a marker of CSCs in both pre-neoplastic and invasive skin cancer [36].

Our data support the previous findings that increased expression of both SOX2 and TP63 are associated with dysregulated cell growth in the epithelial compartment of the cervix. Furthermore, our data suggest identifiable changes in their expression in cervical samples from women with high grade pre-neoplastic disease.

We also showed by flow cytometry that some HR-HPV positive samples from patients with CIN3 have increased numbers of cells that are positive for the cell surface stem cell marker TRA-1-60. Also, these samples demonstrate further enhancement of HCG, TP63 and SOX2 relative to samples from patients with CIN3 that did not show enhanced TRA-1-60. This indicates that samples from patients with CIN3 contain detectable cancer stem cell markers. SOX2, HCG and TP63 mRNAs were also shown to be upregulated in cervical samples with HR-HPV+ve high grade dyskaryosis and SOX2 and TP63 immunohistochemistry identified cancerous cells, in HR-HPV+ve biopsies, from women with cervical squamous cell carcinoma. Flow cytometric analysis of numbers of cycling cells in cervical samples also differentiated normal from high grade disease, regardless of the HPV status of the normal samples, indicating that the increase was disease and not simply HPV-infection related. The increase in numbers of cycling cells was seen whether high grade disease was based on histological examination of a biopsy or on cytology only. This indicates that this testing modality might be applicable in a wider screening context as a laboratory triage test for significant disease. Although there are disturbances in cell cycle control associated with cervical cancer [45], it has been reported that the vast majority of cases are diploid [46]. We found no evidence of aneuploidy in the LBC samples tested from HR-HPV+ve women with high grade disease.

TRA-1-60 is a carbohydrate epitope of the cell surface sialylated keratan sulfate proteoglycan podocalyxin [18], [47] found in human stem cells [18], cancer stem cells [48] and embryonal cancers [49]. Podocalyxin prevents cell adhesion and promotes metastasis. Overexpression is associated with poor prognosis in a number of cancers including breast [50], ovarian [51], renal [52] and uterine [53] cancers. Although podocalyxin has not previously been reported in cervical cancer, it interacts with a protein, ezrin [54], which has been reported to be upregulated in HR-HPV associated cervical lesions where it was found to co-localise with p16inc [55]. Furthermore ezrin overexpression has been reported in cervical cancer biopsies [56]. Podocalyxin has been shown to increase metastatic potential of tumour cells, in vitro, through its interaction with ezrin [57].

We chose to stain SOX2 protein in these biopsies as it has been reported to be upregulated in human cervical cancer stem cells [58] and in SCCC [44]. Normal cervical squamous epithelium showed basal cell staining and all SCCC biopsies showed similar staining of tumour cells, but not stromal cells. Although the SOX2 protein is normally confined to the nucleus, we found that in the cancer cells, there was both nuclear and cytoplasmic expression, which may indicate abnormal accumulation of the protein in tumour cells. TP63 has also been shown to be expressed in cervical cancer [59] and interacts with SOX2 in squamous cell carcinomas [26] we also stained for TP63 protein in parallel sections from the same biopsies. Our results show that SOX2 and TP63 proteins are found in the nucleus of most tumour cells in HR-HPV+ve SCCC biopsies. To our knowledge this is the first time SOX2 and TP63 have been evaluated in the same series of cases with this disease.

Our results indicate that detection of cancer stem cell associated proteins by flow cytometry or western blotting and/or flow cytometric detection of numbers of cycling cells may be useful laboratory tests for triage of HR-HPV+ve cervical samples for significant cervical disease. They further indicate that SOX2 and TP63 immunohistochemistry may have value in diagnosis of HR-HPV related SCCC. It is not possible to follow up any of the patients in this study but it would be of obvious interest in the future to determine the longterm outcome of patients with pre-neoplastic lesions whose cervical samples show increased CSC marker expression and/or numbers of proliferative cells.
